# Sex for food: a transactional sex reaction to the COVID-19-induced rise in the prevalence of hunger among in-school female adolescents in Ogun State, Nigeria

**DOI:** 10.11604/pamj.2023.44.177.35687

**Published:** 2023-04-14

**Authors:** Kolawole Sodeinde, Oluwafolahan Sholeye, Olusegun Adebisi, Adebola Omotosho, Adedotun Sodeinde, Olumide Abiodun

**Affiliations:** 1 Department of Community Medicine, Babcock University, Ilishan, Ogun State, Nigeria; 2 Department of Community Medicine and Primary Care, Olabisi Onabanjo University, Sagamu, Ogun State, Nigeria; 3 Department of Community Medicine and Primary Care, Federal Medical Centre, Abeokuta, Ogun State, Nigeria; 4 Department of Community Medicine, Babcock University Teaching Hospital, Ilishan-Remo, Ogun State, Nigeria; 5 Department of Educational Management, Tai Solarin University of Education, Ogun State, Nigeria

**Keywords:** COVID-19, female adolescents, food security, transactional sex

## Abstract

**Introduction:**

COVID-19 has doubled the proportion of people with acute hunger globally and Nigeria is categorized as having a “severe” form of hunger. Vulnerable groups responding more to hunger stressors include the female adolescents who transact sex for both survival and luxury purposes. This study assessed the engagement in transactional sex to obtain food among female adolescents in Ogun State, Nigeria.

**Methods:**

this descriptive cross-sectional research was conducted among 668 in-school female adolescents that were selected through a multistage sampling method. Data were collected with a structured interviewer-administered questionnaire. Food security was assessed using a standard tool while engagement in transactional sex was elicited through self-report. Data analysis was done using Statistical package for the social sciences (SPSS) version 25. Logistic regression was used to find factors associated with transactional sex. P< 0.05 was taken as statistically significant.

**Results:**

the mean age of the respondents was 15.18±1.35 years. More than two-fifths (41.5%) were staying in households without food security. Less than one-fifth (15.3%) were engaging in transactional sex. Having emotional stress (OR = 4.33; 95 CI: 1.37-13.63), mother´s education (OR =0.163; 95% CI: 0.057- 0.461), having sexual partner (OR = 0.132; 95% CI: 0.047-0.370 and missing classes (OR 3.35, 95% CI: 1.084-10.331) were significant predictors of engaging in transactional sex among female adolescents.

**Conclusion:**

transactional sex is commoner among female adolescents who were missing classes, had sexual partners, and were emotionally stressed. There is a need for intervention programs to protect the female adolescent, delay sexual debut and improve academic performance among them.

## Introduction

Adolescents are people aged between the ages of 10 and 19 years. Globally, about 1.2 billion people fall in this age category making a total of 16% of the world´s population [1]. The period is categorized into early, mid and late adolescence [2]. About a third of the sub-Saharan African population comprises young people and adolescents [3]. This is the only region in the world that has a population of adolescents and young people constantly experiencing extensive growth [4]. Population projections place the number of youth aged 10-24 years to be 436 million by the year 2025 [5]. These individuals are known for fast physical growth and sexual maturation. However, the need to experiment with new things such as drugs and sex also characterizes this period. Such high-risk behaviors include transactional sex (TS) among other things [6]. Transactional sex is defined as the act of engaging in heterosexual intercourse in exchange for financial gains and/or material possessions [6]. According to Stoebenau [7] TS is a non-marital, non-commercial sexual affair that is driven by the supposition of exchange of material help and other forms of benefits for sex. The distinction between TS and sex work can be vague in some instances and there may be an evolution from the former to the latter. Unlike sex work, TS is usually characterized by some level of relationship and those concerned distinguish their act from typical sex work [8]. Friends, associates, and dating websites have been postulated to be responsible for the initial interactions between those engaged in TS, and sellers and buyers of TS often know one another before they trade sex [9]. Besides, the possibility of transition of TS to sex work, a thin line also exists between TS and sexual exploitation. On the side of the male partner, exploitation may exist when there is power inequity in the relationship as fueled by several socio-economic and socio-demographic factors [10]. It may also occur when the man does not fulfill his part of the bargain or when he forcefully has sex with the female counterpart. On the part of the female partner, exploitation may occur when she takes the material or cash but refuses sex or when she requests a large amount of money [10].

Transactional sex (TS) is on the rise among adolescents and young people, and it seems to be endorsed by parents and peers alike [11]. Transactional sex (TS) among this age group is associated with several negative outcomes such as delinquency, physical or sexual violence, psychological issues, various physical illnesses, sexually transmitted diseases [12], and HIV [13]. The prevalence of TS among young people has been documented to vary in different climes. It is less common in high-income countries as compared to lower- and middle-income countries [14]. In the latter, there is also a well-pronounced disparity in the prevalence of TS; ranging from 2.1 to 30.4% among females in sub-Saharan Africa [15]. Moreover, evidence shows TS as a commoner phenomenon among boys in developed countries and girls in under-developed countries [9]. In Nigeria, the culture of postponing sexual activities among adolescent females till after the wedding has been challenged as TS among girls has increasingly gained popularity [16]. Evidence has shown that the prevalence of hunger has risen slowly since 2015 after an initial steady decline over decades. It is estimated that 8.9% of the global population or about 690 million people were hungry by the beginning of 2020 with the COVID-19 pandemic likely to have doubled the proportion of people who suffer acute hunger by the end of the year [17]. In 2020, Nigeria had a global hunger index of 29.2 putting Nigeria at a level of hunger that is classified as “serious” [18]. Several benefits derived from TS vary among different classes of women. According to Tade *et al*. [16], undergraduate adolescent girls and young women engage in TS for luxury properties including phones, cars, and employment after school. However, other authors have posited less expensive gains as reasons adolescent girls transact sex. For instance, Wojcicki postulated that meeting basic sustenance needs is the motive for engagement in TS [19]. According to Atwood, adolescent girls engage in TS for food and some other sustenance needs [11]. This may be because adolescent girls experience hunger more than their male counterparts and respond more to the stressors of food insecurity [20] which has particularly increased with the COVID-19 pandemic [17]. Little has been done on the issue of TS among adolescents and the subject only started gaining research interest recently [21]. Moreover, studies on TS for the simple purpose of meeting nutritional needs and satisfying hunger among female adolescents are not popular. This study aimed to assess the engagement in TS to obtain food among female adolescents in Ogun State, Nigeria.

## Methods

**Study area and study design:** this descriptive cross-sectional study was conducted in March 2021 among female adolescents in secondary schools in Ogun State, Nigeria. The State is bounded in the north by Osun and Oyo States, in the south by Lagos State, Ondo State in the east, and the Republic of Benin in the west [22]. The projected population of the State in 2016 was 5,217,700 [23]. There are twenty (20) local government areas (LGAs) in Ogun State, with each LGA headed by an executive chairman. The State is divided into three senatorial districts, eight geopolitical zones, and nine federal and 26 State constituencies. Ogun State has a total of 1,997 secondary schools, comprising 510 publics and 1,487 private schools, with a total population of about 742,633 students. It has two federal tertiary hospitals, one State tertiary health facility, one private tertiary health facility, 39 public secondary health facilities, 450 primary health facilities, and 904 private health facilities [24].

**Study population and sample size estimation:** participants were female adolescents aged 10-19 years old who had spent at least 12 months in the different selected schools. A minimum sample size of 380 was calculated by using the Cochran formula [25]


$$n=\frac{Z^{2}pq}{d^{2}}$$

with n being the desired sample size, z is the normal standard deviation taken at 1.96 (for 95% confidence level), P is the prevalence of the characteristic of the study, q is 1.0 ? p and d is the desired degree of accuracy taking as 0.05. The prevalence (p) was taken as 54.6% which was the prevalence of adolescent school girls involved in transactional sex in an Ethiopian study [6]. A 40% non-response rate was added to the sample and the minimum sample size became 633.3. However, a total of 668 female adolescents were interviewed in this study.

**Sampling method, data collection tools, and techniques:** multi-stage sampling method was used to select the participants for the study. There are three senatorial districts in Ogun State. In stage; i) simple random sampling was used to select one senatorial district which turned out to be Ogun East Senatorial District. The senatorial district is made up of 9 local governments. In stage; ii) simple random sampling was used to select one local government area (LGA). Sagamu LGA was selected. In stage; iii) simple random sampling was used to select 4 wards out of the 15 political wards in the LGA. Then public secondary schools in the wards were selected. Where there was more than one public secondary school in a ward, simple random sampling was used to select one secondary school. In stage; iv), systematic random sampling was used to select participants in each school using the proportionate to size technique. Data were elicited from the respondents by using a structured interviewer-administered questionnaire. The instrument was constructed from a review of literature on transactional sex and sexual behaviors among teenagers and also on food security. The questionnaire was assessed for content and face validity and pre-tested on 60 female adolescents in Lagos State which is in the same geopolitical zone as the State under study. Corrections were then made to the questionnaires. The questionnaire sought information on respondents´ socio-demographic characteristics, behavioral characteristics, and household food security.

### Study measures

**Sex for food:** any of the participants who have ever had sex in exchange for food or money to purchase food.

**Had sexual partner:** any participant who has ever had a sexual partner different from and before or concurrently with the transactional sex partner.

**Emotional stress:** for this study, this includes any adolescent who had at least one of the following experiences in the past 4 weeks; a) have been bullied; b) have engaged in a fight; c) have felt lonely.

**Smoking cigarette:** recently smoking any quantity of cigarettes within the last month [26].

**Alcohol intake:** recently take any quantity of alcohol within the last one month [26].

**Data management and analysis:** the questionnaires were cross-checked for errors and cleaned. The analysis was done using Statistical Package for Social Sciences (SPSS) version 25.0. Composite variables (aggregate scores) for food security were computed from items on the questionnaire. Nine items were assessed for food security giving a total score of 9. The items/questions asked about unavailability and inadequacy of food/feeding in the adolescents´ household. Those who scored 2 and below were considered to have come from households with food security while those who scored 3 and above were considered to have come from households with food insecurity. Engagement in transactional sex was assessed by asking if participants had ever had sex in exchange for food by providing the options “Yes” and “No”. Data analyzed were presented as frequency tables. Data summarization was done using mean, standard deviation, and proportions. Association between categorical variables was done using Chi-squares. Logistic regression analysis was used to test factors responsible for transactional sex after controlling for confounding. These factors were the statistically significant variables (p-value <0.05) in the bivariate analysis. The level of significance was set at a 95% confidence interval with a P value=0.05.

**Ethical Approval:** “Approval for this research was obtained from the ethics committee of the Ogun State Ministry of Health (HPRS/381/405). Approval was also obtained from the State Ministry of Education through the office of the Principal-General for Remo zone and from the respective school authorities, through the principals. Verbal and written informed consent was obtained from the respondents aged 16 and above. Parental consent was obtained for younger adolescents and assent was also obtained from them. Strict confidentiality of all information and results of findings was ensured throughout the study.

## Results

Table 1 shows the socio-demographic characteristics of the respondents. Three-quarters (75.0%) of the participants were in the mid-adolescent stage (14-16 years) while one-tenth (9.9%) were in the early adolescent stage (10-13 years). The mean age was 15.18±1.35 years. Almost all (97.6%) of the participants were single and just over half (52.2%) were in senior secondary class 1 (SS1). A little below three-fifth were staying with both parents (57.8%) and lived in a household with food security (58.5%). Table 2 shows the behavioral characteristics of the adolescents. A little over one-tenth (11.2%) missed at least 3 classes in the past two weeks before the survey. A few of them were currently taking alcohol (7.8%) and smoking cigarettes (6. 9%). A history of sexual partners was reported among 102 (15.3%) of the participants. Table 3 shows that there was a statistically significant association between marital status (<0.001), history of sexual partner (<0.001), having missed at least three classes in the last two weeks (<0.001), emotional stress (p<0.001), mother´s education (0.002) and household food security (0.001) with transacting sex for food. A higher proportion of those who were married, divorced, or separated, classified as “Not single” (23.5%) were involved in transactional sex as compared to only 3.1% of those who were single (never being married). A higher proportion of those with a history of sexual partners (10.8%) were involved in transactional sex as compared to only 2.3% of those who never had a sexual partner. A higher proportion of those who had missed at least three classes in the last two weeks (10.7%) were engaging in transactional sex compared to only 2.7% of those who did not miss three classes or more in the last two weeks. A higher proportion of those who had a recent history of emotional distress (7.4%) was engaged in sex in exchange for food as opposed to only 1.3% of those who did not have emotional distress were engaged in sex in exchange for food. A higher proportion of adolescents whose mothers had tertiary education (8.1%) had sex for food as compared to only 2.4% of those whose mothers did not have tertiary education who were engaged in sex for food. A higher proportion of adolescents who were from households with food insecurity (6.5%) were engaged in sex in exchange for food as compared to only 1.5% of those who were from households with food security that had sex for food. Table 4 shows that the mother´s education (OR 0.163, 95% CI: 0.057-0.461), having a history of sexual partner (OR 0.132, 95% CI: 0.047-0.370), having a recent history of emotional stress (OR 4.33, 95% CI: 1.373- 13.632) and having missed 3 or more classes in last two weeks (OR 3.35, 95% CI: 1.084- 10.331) remained significant predictors of having sex in exchange for food among female adolescents.

**Table 1:** socio-demographic characteristics of respondents (N=668)

Variable	Frequency	Percentage
**Age (Years)**		
10-13	66	9.9
14-16	501	75.0
17-19	101	15.1
**Marital status**		
Single	651	97.6
Married	11	1.6
Divorced	3	0.4
Separated	3	0.4
**Class**		
J.S.S 3	12	1.8
S.S.S 1	349	52.2
S.S.S 2	307	46.0
**Mother’s educational status**		
No formal education	23	3.4
Primary	94	14.1
Secondary	415	62.1
Tertiary	136	20.4
**Mother’s occupation**		
Housewife	121	18.1
Unskilled	284	42.5
Semi-skilled	150	22.5
Skilled	113	16.9
**People living with**		
Mother only	154	23.1
Father only	72	10.8
Both parents	386	57.8
Siblings	27	4.0
Extended family	25	3.7
Boarding house	2	0.3
Guardian	2	0.3
**Number of people living together**		
≤3	106	15.9
4-6	452	67.7
7-9	97	14.5
≥10	13	1.9
**Household food security**		
Yes	391	58.5
No	277	41.5

Mean age: 15.18±1.35

**Table 2:** behavioural characteristics of respondents

Variable	Frequency	Percentage
**Missed ≥ 3 classes in last two weeks**		
Yes	75	11.2
No	593	88.8
**Currently, take alcohol**		
Yes	52	7.8
No	616	92.2
**Currently, take cigarette**		
Yes	46	6.9
No	622	93.1
**History of sexual partner**		
Yes	102	15.3
No	566	84.7
Emotional stress		
Yes	271	40.6
No	397	59.4

**Table 3:** respondents’ characteristics and bivariate relationship with sex for food

Variable	History of sex for food		χ2	p-value
	Yes n (%)	No n (%)		
**Age (years)**				
10-13	3 (4.5)	63 (95.5)		
≥ 14	21 (3.5	581 (96.5)	0.192	0.66
**Class**				
Junior class	0 (0.0)	12 (100.0)		
Senior class	24 (3.7)	632 (96.3)	0.455	0.50
**Marital status**				
Single	20 (3.1)	631 (96.9)		
Not single	4 (23.5)	13 (76.5)	20.017	< 0.001
**Person(s) living with**				
Both parents	11 (2.8)	375 (97.2)		
Not both parents	13 (4.6)	269 (95.4)	1.458	0.23
**Number of people living together**				
≤ 5	15 (3.6)	400 (96.4)		
>5	9 (3.6)	244 (96.4)	0.001	0.97
**History of sexual partner**				
Yes	11 (10.8)	91 (89.2)		
No	13 (2.3)	553 (97.7)	17.97	< 0.001
**Currently taking alcohol**				
Yes	4 (7.7)	48 (92.3)		
No	20 (3.2)	596 (96.8)	2.74	0.10
**Currently smoking cigarette**				
Yes	1 (2.2)	45 (97.8)		
No	23 (3.7)	599 (96.8)	0.287	0.59
**Missed ≥ 3 classes in last two weeks**				
Yes	8 (10.7)	67 (89.3)		
No	16 (2.7)	577 (97.3)	12.205	<0.001
**Emotional stress**				
Yes	20 (7.4)	251 (92.6)		
No	5 (1.3)	392 (98.7)	15.465	<0.001
**Mother’s education**				
Tertiary	11 (8.1)	125 (91.9)		
Non-Tertiary	13 (2.4)	519 (97.6)	9.963	0.002
**Mother’s occupation**				
Housewife	3 (2.5)	118 (97.5)		
Working	21 (3.8)	526 (96.2)	0.529	0.47
**Household food security**				
Food secure	6 (1.5)	385 (98.5)		
Food insecure	18 (6.5)	259 (93.5)	11.533	0.001

**Table 4:** respondents’ characteristics and multivariate relationship with sex for food

Variable	Odd’s ratio	Confidence interval	p-value
**Marital status**			
Single	0.230	0.042- 1.259	0.09
Not Single	1.00		
**Mother’s education**			
Tertiary	1.00		
Non-Tertiary	0.163	0.057- 0.461	0.001
**History of sexual partner**			
Yes	1.00		
No	0.132	0.047- 0.370	<0.001
**Emotional stress**			
Yes	4.33	1.373- 13.632	0.012
No	1.00		
**Household food security**			
Food Secured	0.459	0.160- 1.319	0.148
Food In-secured	1.00		
**Missed ≥3 classes in the last two weeks**			
Yes	3.35	1.084- 10.331	0.036
No	1.00		

## Discussion

Young women engage in transactional sex for several reasons, but the common meeting point is the intention to meet essential needs and, in some instances, obtain luxurious material possessions [10]. The patriarchal nature of many African societies also influences sexual relationships whereby men provide financial and material support for women who are expected to return the favor with sex. Hence, some societal norms lend support to TS in which adolescents and young women engage in TS and expect financial or material support from their male partners [27]. Young women with restricted privileges have also been postulated to upgrade their social class in some instances by engaging in TS [28]. Sustainable development goal 2 preaches zero hunger by the year 2030. However, this appears like a mission impossible as a large proportion of the world is currently facing hunger. This has been aggravated by the currently ravaging COVID-19 disease and the number of people affected by hunger globally is projected to exceed 840 million by the year 2030 with the current trends [17]. Our study also observed a high proportion of households with food insecurity and this may not be associated with the current pandemic. Contrary to our finding, a study by Poole Di Salvo *et al*. [29] reported a much lower prevalence of food insecurity in households with adolescents during the pre-COVID-19 days and this further corroborated the impact of the viral outbreak on food availability in households. Moreover, our study observed a high proportion of transactional sex in exchange for food among female adolescents in households with food insecurity as compared to those from households without food insecurity. This affirms the postulations from several authors that female adolescents often use TS to meet their various needs [16,19,30] which may be as basic as feeding [11] in which case, sex is sold for survival. Furthermore, it is opined that girls get hungry more easily [20] perhaps because of increased nutritional requirements for various physiological functions [31,32]. They are also more proactive in offering solutions to hunger pressures [20].

In this current study, the history of having a sexual partner and having ever been married both have direct associations with engagement in transactional sex for food. This suggests that adolescent girls who have been exposed to sex one way or the other had fewer constraints as to having sex in the other to offer a solution to the unavailability of food. Also, the erotic experience from previous intercourses may have become rather enjoyable or even addictive to the girl who would crave further experiences irrespective of the circumstances of such exposures. In a similar vein, several studies posit that a direct relationship exists between previous sexual exposure either voluntary or involuntary, and transactional sex. According to Krisch [9], promiscuity and involvement in various risky sexual behaviors are important correlates of TS among female adolescents. Ahrens [33] postulated that a history of rape and other forms of sexual molestation significantly increased the odds of transacting sex among female adolescents. Okigbo *et al*. [34] reported that early coitarche increased period of sexual involvement, history of sexual violence, and having multiple sexual partners were all positively correlated with involvement in TS among young females. In a study on TS and HIV risk among female adolescents in Ethiopia, Dana *et al*. [6] reported that over half of the sexually active participants were involved in TS, and about one-fifth of them were nursing the idea. It has been documented that many adolescents who live in food in-secured homes are more vulnerable to psychosocial issues and mental health [35]. In our study, having recent emotional stress had a significant association with being engaged in TS among female adolescents. With the high prevalence of food insecurity in our study discussed earlier it, therefore, appears that a linear relationship may exist between food insecurity, emotional stress, and involvement in TS as female adolescents who live in homes where their daily nutritional needs cannot be met may battle with emotional issues and opt for TS as a way out. In post-conflict Liberia [34], three out of four young females had transactional sex and this may offer credence to this possible association. Post-conflict era is usually characterized by food shortage and post-traumatic/emotional stress, therefore female adolescents who are by nature vulnerable [36] may seek succor through TS. However, this association discussed may not be as linear as it is presented as getting involved in TS itself can cause the young adolescent girl some level of emotional stress especially when the female has a sense of being exploited by the male partner [10]. According to Furman [36], relationships can challenge self-confidence among young women. We found increased odds of engaging in TS among female adolescents who missed three or more classes in the last two weeks. Less commitment to school activities may offer the adolescent opportunity to engage in TS and other extracurricular activities. Findings from previous studies lend support to this assertion. According to Kirby [37], adolescents who are fully involved and attached to school engage in less sexual activities. According to him, there are specific school programs that help the adolescents to be committed to school, reduce drop-outs and delay the onset of sexual involvements [37]. Likewise, similar studies in Kenya [38] Uganda [39] and Italy [40] posited a negative relationship between school commitment and engagement in TS.

**Limitation of the study:** while to the best of our knowledge, we see this study as the only one in Nigeria that has assessed the engagement in transactional sex among female adolescents specifically to get what to eat especially during the ravaging COVID-19 pandemic which has aggravated the level of hunger globally. However, this study does have some limitations. The cross-sectional nature does not allow proper measurement of temporal associations between the measured independent variables and transactional sex. Besides, information was obtained through self-report of the various exposure variables and there may have been under and/or over-reporting. Future areas of study may include cohort studies to show determinants of transactional sex among female adolescents.

## Conclusion

This study highlights factors that contribute to engagement in transactional sex in exchange for feeding needs during the COVID-19 pandemic. Poor academic commitment, previous involvement in different forms of sexual activities, mothers’ education, and emotional stress all have increased odds of engagement in transactional sex among female adolescents. Household food insecurity was also associated with increased TS, though it was not significant after controlling for confounders in multivariate analysis. Our findings, therefore, lend a voice to the need for intervention policies and programmes that will protect the right of the girl child as she is shielded from all forms of assault, improve the female adolescent commitment to academic activities, delay sexual debut among them and improve the agriculture system for better food productivity while empowering parents and caregivers to be able to provide same for the household.

### What is known about this topic

- *Adolescents are typically involved in high-risk behaviours such as transactional sex;*
- *Transactional sex among adolescents is not only to acquire luxurious items but also to for sustenance such as getting food to eat among hungry youths;*
- *COVID-19 has increased the prevalence of hunger globally*.

### What this study adds

- *Female adolescents who have history of sexual partners are more likely to transact sex for food;*
- *Truancy in school has direct relationship with transacting sex for food among female adolescents;*
- *Female adolescents who pass through stress are also likely to transact sex for food*.
